# Prevalence of Hypertension in Chinese Cities: A Meta-Analysis of Published Studies

**DOI:** 10.1371/journal.pone.0058302

**Published:** 2013-03-06

**Authors:** Yu-Quan Ma, Wen-Hua Mei, Ping Yin, Xiao-Hui Yang, Sana Kiani Rastegar, Jian-Dong Yan

**Affiliations:** 1 Zhuhai People’s Hospital, Affiliated Zhuhai Hosptal of Jinan University, Zhuhai, Guangdong Province, China; 2 Department of Epidemiology and Health Statistics, Public Health School, Tongji Medical College, Huazhong University of Science and Technology, Wuhan, Hubei Province, China; Tehran University of Medical Sciences, Iran (Republic of islamic)

## Abstract

**Background:**

Hypertension has been recognized as a health concern for developing countries. However, there are no current nationwide surveys on the prevalence of hypertension in China (the latest nationwide survey was ten years ago). The goal of this study was to estimate the pooled prevalence of hypertension in Chinese cities.

**Methods:**

We systematically reviewed published epidemiologic studies on the prevalence of hypertension in Chinese cities through meta-analysis. We searched for studies published between January 2002 and June 2012 using PubMed and two Chinese electronic publication libraries. The keywords ‘hypertension’ and ‘prevalence’ were used. Before pooling prevalence of hypertension, all raw prevalence data was age adjusted to the 2010 China standard population. Prevalence estimates were stratified by sex and geographic area.

**Results:**

27 studies were identified with of a total of 195,027 study participants. The overall pooled prevalence of hypertension was 21.5% (19.4%, 23.6%). Subgroup analyses showed the following results north 25.8% (21.6%, 30.0%), south 20.4% (18.6%, 22.2%); male 22.2% (19.3%, 25.1%), female 19.9% (17.6%, 22.1%); large cities 18.9% (15.7%, 22.1%), medium-sized cities 24.6% (19.9%, 29.4%), small cities 20.6% (17.5%, 23.7%); study years in 2002–2006, 21.9% (18.9%, 24.8%), and study year in 2007–2011, 20.6% (17.3%, 23.9%).

**Conclusions:**

Comparing data from several previous national hypertension surveys, the prevalence of hypertension is higher in cities than the Chinese national average. Subgroup studies also found a higher prevalence of hypertension in northern cities and among males. Also, the prevalence of hypertension in medium-sized and small cities is likely to increase faster than in large cities.

## Introduction

Hypertension is the most common risk factor for cardiovascular diseases (CVD) [1]. Hypertension has been recognized as a global, chronic, non-communicable disease and as a “silent killer” due to its high mortality rates and lack of early symptoms. One-quarter of the world’s adult population is hypertensive and this figure is likely to increase to 29% by 2025 [2]. The absolute prevalence of hypertension in developing countries is 22.9% compared with 37.3% in developed countries [2]. Unfortunately, the prevalence of hypertension is growing rapidly in developing countries which are undergoing epidemiological transitions, economic improvement, urbanization and longer life expectancy [3]. Kearney et al. [1] estimated that by 2025, the number of individuals with hypertension in developing countries will be approximately 1.17 billion, representing almost three-fourths of the world’s hypertensive population. A cross-sectional study from 2002 estimated that nearly 18% of Chinese people age 15 years and older, corresponding to 177 million people, were hypertensive [4].

As a result of policy reform many people’s lifestyle in many parts of China has become more westernized. In cities, individuals consume a diet high in salt, calories, saturated fat and low in fruits and vegetables. Several studies have hypothesized that these changes may contribute to a higher prevalence of hypertension in urban populations when compared to rural populations [5]–[8].

“Prevention first,” is the best and most important strategy for China. This strategy requires a sensible plan of action for prevention and improving current policies against hypertension. However, there are no current nationwide studies on the prevalence of hypertension in Chinese cities; the latest nationwide investigation is now ten years old [9]. Therefore, we performed this meta-analysis to systematically review the findings of all current studies and estimate the overall prevalence of hypertension in Chinese cities.

## Materials and Methods

### Literature Search Strategy

We searched all published articles for epidemiologic studies on the prevalence of hypertension between January 2002 and June 2012 in three electronic databases: CNKI, WANFANG and PUBMED. These results were supplemented by several useful references cited in the key articles. Articles were identified with search strategy “hypertension” AND (“prevalence” OR “epidemiologic studies”) in all databases. Two authors (YQ Ma and WH Mei) screened the titles and abstracts and reviewed the full-text of the eligible articles. Studies in the areas of Hong Kong, Macao and Taiwan were excluded due to cultural differences from mainland China.

### Criteria for Inclusion and Exclusion

We included all epidemiologic studies which reported the prevalence of hypertension among Chinese residents over 15 years of age. Enrollment years of the studies used were between 2002 and 2011. Only studies that used random sampling or census data to find participants were used. All source studies were original research written in English or Chinese and contained the minimum information necessary to calculate pooled analysis of prevalence (number of the subjects and number of hypertension events). Studies were included if they explicitly defined hypertension as an average systolic BP (SBP)≥140 mmHg, an average diastolic BP (BP)≥90 mmHg, or self-reported current treatment for hypertension with an antihypertensive medication as defined by the Seventh Annual Report of the Joint National Committee (JNC 7) [10].

We excluded studies if the participants were limited to a particular occupation, population, age group, community group or location. We excluded studies whose age-specific prevalence of hypertension was not reported. We also excluded studies that utilized nonrandom sampling or non-JNC 7 standards (e.g. self-reported hypertension without measuring blood pressure).

### Data Extraction

A standardized data abstraction form was designed to capture and code all relevant study-level information required for analysis. Two investigators selected the studies, extracted the data independently, cross-checked them and resolved disagreements by consensus. For all included studies, we recorded the following information: author, year of publication, year of investigation, city, sampling method, data collection method, diagnostic criteria of hypertension, age of subjects, number of subjects, number of people with hypertension, and age-specific prevalence of hypertension.

### Statistical Analysis

To estimate pooled age-standard prevalence of hypertension, all raw prevalence data was age adjusted to the 2010 China standard population [11]. According to the expected heterogeneity across studies, a random-effects model was used to calculate pooled age-standard prevalence and calculate 95% confidence intervals (CIs). Statistical heterogeneity was evaluated with the Cochran chi-square (χ^2^) and quantified with the I^2^ statistic (low is <25%, moderate 25–50%, high >50%) [12]. In order to deal with heterogeneity and perform secondary analysis, subgroup analysis was necessary. Subgroups were defined as differences in sex, year range of investigation (every two years), and geographical region differences (south and north). Publication bias was evaluated by testing for funnel plot asymmetry, Begg’s Test and Egger’s test. Significance was set at a P value of less than 0.05. All statistical calculations were made using the Stata® Statistical Software Package, Version 11.0 (StataCorp LP, College Station, Texas, USA) and RevMan 5.1 Software (Cochrane Collaboration, London, UK).

## Results

**Figure 1:**
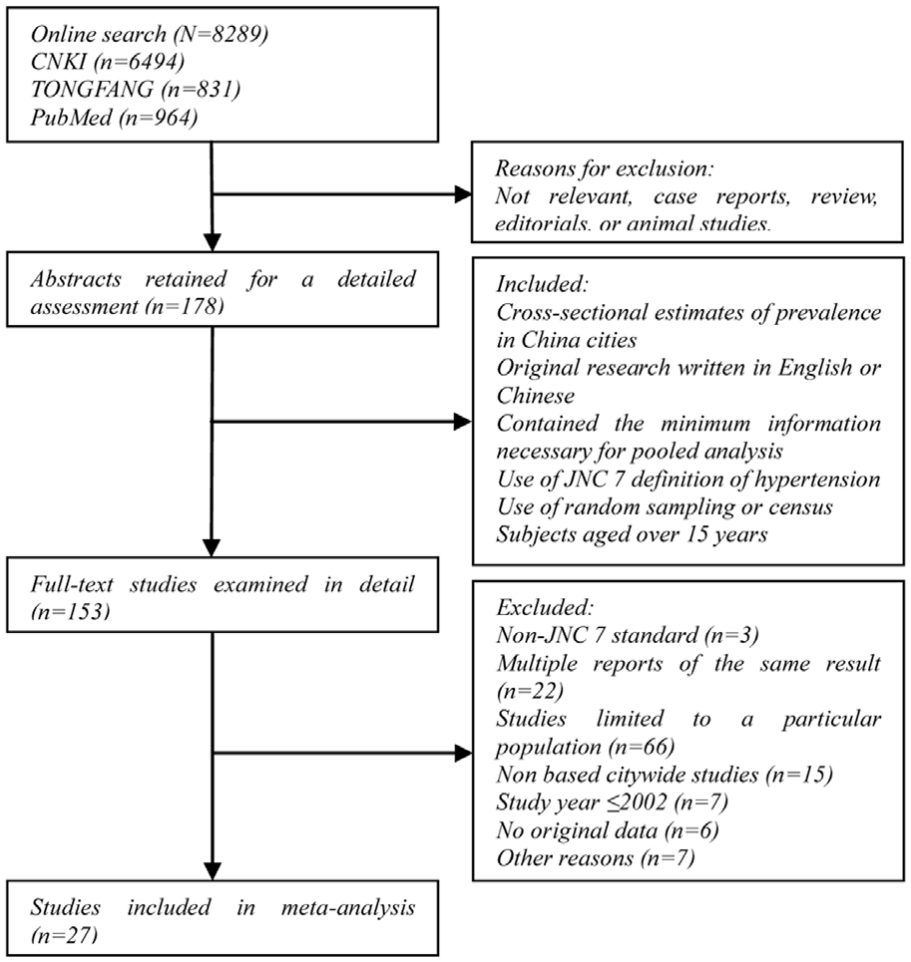
Flow diagram of the study selection process. As shown, our initial searches yielded 8,289 citations: 6,494 from CNKI, 831 from TONGFANG, and 964 from PubMed/MEDLINE. After screening titles and abstracts, 125 studies were considered potentially eligible and retrieved in full text. Of these, 97 studies were subsequently excluded because they did not satisfy the inclusion criteria. Thus, 27 fully eligible studies were identified.

After an initial search identified 8,289 studies, application of inclusion and exclusion criteria, 27 studies were selected [13]–[39]. These 27 studies contained of a total of 195,027 subjects determined to be suitable for meta-analysis (Figure 1; Table 1). Most excluded studies focused on a specific population. Furthermore, many studies from the initial search duplicated existing results. Of the 27 identified studies, nine studies [13], [14], [17], [19], [22], [29], [30], [36], [38] did not report age-specific data according to sex, but had no effect on overall analysis. In summary, age was limited to 15 year olds and above in 20 studies [13], [15]–[29], [31], [33], [37], [39]; 15–69 in four [32], [34]–[36]; and 15–74 in three [14], [30], [38]. Twenty-two studies [14]–[24], [26]–[29], [31]–[35], [37], [39] were from cities in southern China and five [13], [25], [30], [36], [38] were from cities in northern China. Seven studies [13], [14], [27], [34], [35], [37], [39] were from large cities, nine were from medium-sized cities [15], [16], [20], [25], [26], [28], [29], [36], [38], and 13 [17–19, 21–24, 39–33] were from small cities. Nineteen studies enrolled participants between 2002 and 2006 [13]–[31] and eight studies enrolled participants between 2007 and 2011 [32]–[39].

**Table 1:** Summarized information of the studies included in meta-analysis.

First author & year published	Study year	Study city (city size^a^/geographic areas^b^)	Population^c^	Age	BP measurement frequency	Sample size(male/female)	Prevalence (male/female) (%)	Age-standard prevalence (male/female) (%)
Jiao SF, et al. (2005)	2002	Beijing (A/N)	NR^d^	≥15	2	24725(12018/12707)	26.4(27.0/25.8)	24.5(NA^d^/NA^d^)
Wu WQ, et al. (2006)	2002	Shanghai (A/S)	5 years PP	15–74	3	8793(3273/5520)	29.4(32.1/27.9)	18.2(NA^d^/NA^d^)
Liu XF, et al. (2007)	2002	Chongqing (B/S)	NR^d^	≥15	2	4260(1787/2473)	20.7(21.5/20.2)	14.9(15.6/16.3)
Fang SY, et al. (2004)	2003	Hangzhou (B/S)	GP	≥15	3	1070(476/594)	37.5(39.1/36.2)	24.9(25.7/24.1)
Cui MY, et al. (2004)	2003	Huzhou (C/S)	PP	≥15	3	1028(457/571)	32.2(33.0/31.5)	24.3(NA^d^/NA^d^)
Gu WL, et al. (2004)	2003	Jiaxing (C/S)	GP	≥15	3	1277(513/754)	29.8(33.3/27.7)	23.0(25.6/21.3)
Xu ZX, et al. (2004)	2003	Jinhua (C/S)	GP	≥15	NR^d^	939(NR^d^/NR^d^)	31.0(NR^d^/NR^d^)	24.3(NA^d^/NA^d^)
Zhang LY, et al. (2004)	2003	Ningbo (B/S)	NR^d^	≥15	3	1162(530/632)	34.8(37.5/32.4)	24.9(28.3/22.1)
Jiang PZ, et al. (2004)	2003	Quzhou (C/S)	NR^d^	≥15	3	1061(391/670)	45.2(50.4/42.4)	25.7(28.7/24.4)
Zhang YF, et al. (2004)	2003	Shaoxing (C/S)	GP	≥15	NR^d^	1038(475/563)	27.3(30.1/24.9)	23.2(NA^d^/NA^d^)
Chen LJ, et al. (2006)	2003	Taizhou (C/S)	PP	≥15	3	1058(478/580)	36.0(32.6/38.8)	23.8(22.7/24.7)
Zhang JH, et al. (2006)	2003	Zhoushan (C/S)	GP	≥15	3	1174(407/767)	28.2(28.0/28.3)	22.1(20.8/23.0)
Li SP, et al. (2007)	2003	Qingdao (B/N)	NR^d^	≥15	NR^d^	10080(4088/5992)	39.9(44.0/37.1)	30.0(33.6/28.2)
Hong Y, et al. (2007)	2003	Wenzhou (B/S)	PP	≥15	3	1034(402/632)	39.0(47.8/33.4)	28.4(36.8/22.9)
Liu WJ, et al. (2007)	2004	Guangzhou (A/S)	NR^d^	≥15	2	23485(11131/12354)	17.2(16.9/17.5)	15.3(14.7/16.0)
Li L, et al. (2007)	2005	Hangzhou (B/S)	NR^d^	≥15	2	1452(591/861)	29.1(30.1/28.5)	25.7(26.6/25.1)
Huang XB, et al. (2009)	2005	Chongqing (B/S)	NR^d^	≥15	NR^d^	5246(2443/2803)	18.5(18.8/18.2)	15.9(NA^d^/NA^d^)
Liu ST, et al. (2007)	2006	Mianyang (B/N)	2 years PP	15–74	3	5875(2755/3120)	29.0(32.3/26.1)	17.6(NA^d^/NA^d^)
Tang XR, et al. (2008)	2006	Jiangmen (C/S)	NR^d^	≥15	3	15018(6457/8561)	13.7(13.3/13.9)	10.0(9.2/10.7)
Chen B, et al. (2011)	2007	Zhuhai (C/S)	6 months PP	15–69	3	961(436/525)	23.1(23.9/22.5)	17.7(19.1/16.9)
Li CL, et al. (2008)	2008	Yibin (C/S)	NR^d^	≥15	NR^d^	26143(13179/12964)	17.4(18.1/15.5)	16.2(17.0/15.3)
Zhou Q, et al. (2010)	2008	Guangzhou (A/S)	6 months PP	15–69	3	6987(3264/3723)	25.5(25.5/25.4)	20.2(20.2/20.0)
Li XJ, et al. (2010)	2008	Shanghai (A/S)	6 months PP	15–69	3	17174(8072/9102)	23.6(24.0/23.3)	15.4(16.3/14.5)
Yan YY, et al. (2010)	2008	Shijiazhuang (B/N)	PP	15–69	NR^d^	7668(3588/4080)	27.1(26.3/27.8)	28.7(NA^d^/NA^d^)
Peng ZR, et al. (2011)	2009	Shenzhen (A/S)	5 years PP	≥15	3	8782(3833/4949)	14.3(16.7/12.4)	15.2(17.4/13.3)
Wang HX, et al. (2010)	2010	Hohhot (B/N)	5 years PP	15–74	NR^d^	1874(758/1116)	32.9(32.2/33.3)	28.3(NA^d^/NA^d^)
Cheng MN, et al. (2012)	2010	Shanghai (A/S)	6 months PP	≥15	3	15663(7889/7774)	31.1(32.0/30.3)	23.3(25.0/21.6)

a According to the universally agreed standard(population size, GDP, et al.), china cities is divided into three categories, including level A cities (large cities), level B cities (medium-sized cities) and level C cities (small cities).

b geographic areas refers to geographic location of china cities, including southern (S: southern) cities and northern (N: northern) cities.

c GP: General population; PP: Permanent population.

d NR: not reported in original paper; NA: not available.

**Figure 2:**
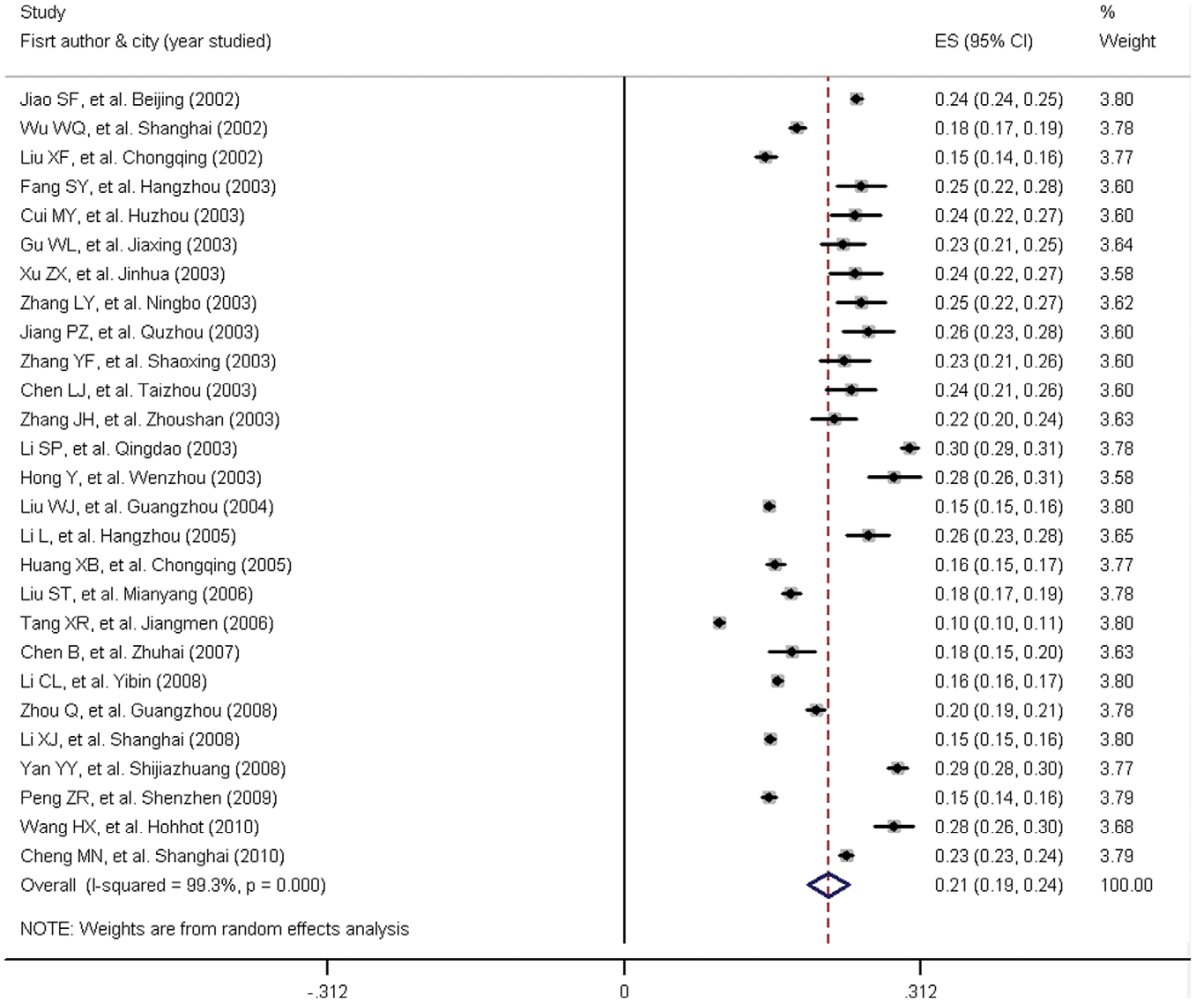
Forest plot of all selected studies. Figure 2 shows unadjusted prevalence estimates (boxes) with 95% confidence limits (bars) for each study selected; pooled prevalence estimates are represented as diamonds in this plot.

Table 1 and Figure 2 show detailed information from the 27 studies selected. The pooled age-standardized prevalence of hypertension was 21.5% (19.4%, 23.6%). Pooled age-standardized prevalence of all subgroups, according to geographical areas, sex, city size and study years are presented in Table 2. The summarized prevalence of northern and southern China were found to be 25.8% (21.6%, 30.0%) and 20.4% (18.6%, 22.2%) respectively. Male and female subgroups were 22.2% (19.3%, 25.1%) and 19.9% (17.6%, 22.1%) respectively. Prevalence in large cities, medium-sized cities and small cities were 18.9% (15.7%, 22.1%), 24.6% (19.9%, 29.4%) and 20.6% (17.5%, 23.7%), respectively. For the first half and last half of the decade covered in this meta-analysis, pooled age-standardized prevalences were 21.9% (18.9%, 24.8%) and 20.6% (17.3%, 23.9%) respectively. Results showed that the pooled age-standard prevalence in northern cities in China was higher than southern cities in China. The prevalence of hypertension in the male population was higher than in females. In addition, the prevalence in medium-sized cities was the highest of the three city size categories. Small city were second and large cities were the lowest. The prevalence was lower in studies conducted between 2007 and 2011 when compared with studies conducted in the first five years.

**Table 2:** Hypertension prevalence according to geographic areas, sex, city size and study years.

Study or Subgroup	No. of Studies	Prevalence^a^ (95% CI) (%)	N	Heterogeneity Test		Publication Bias Test	
				I^2^ (%)	*P*	*P* (Begg’s Test)	*P* (Egger’s Test)
Total	27	21.5(19.4, 23.6)	195027	99.3	0.0031	0.868	0.943
Geographic areas (north)	5	25.8(21.6, 30.0)	50222	99.0	0.0022	0.806	0.819
Geographic areas (south)	22	20.4(18.6, 22.2)	144805	98.8	0.0018	0.215	0.099
Sex (Male)	18	22.2(19.3, 25.1)	63924	98.9	0.0038	0.762	0.767
Sex (Female)	18	19.9(17.6, 22.1)	73907	98.4	0.0022	0.762	0.624
City size (large cities)	7	18.9(15.7, 22.1)	105609	99.4	0.0019	0.548	0.144
City size (medium-sized cities )	9	24.6(19.9, 29.4)	33846	99.0	0.0052	0.348	0.192
City size (small cities )	11	20.6(17.5, 23.7)	55572	98.7	0.0026	0.213	0.058
Study years (2002–2006)	19	21.9(18.9, 24.8)	109775	99.4	0.0043	0.889	0.806
Study years (2007–2011)	8	20.6(17.3, 23.9)	85252	99.3	0.0022	0.711	0.882

Abbreviation: N, total number of subjects from the included studies.

a before the pooled prevalence was estimated, all raw prevalence data was age adjusted to the 2010 China standard population.

Table 2 shows information regarding heterogeneity and publication bias. We noted significant heterogeneity within studies and subgroups (*P*<0.000, I^2^ = (99.2–99.7)).

## Discussion

In our systematic review and meta-analyses of hypertension prevalence, 27 epidemiological studies from 22 cities were selected. These studies covered nine provinces and municipalities in China. All data was collected between 2002 and 2011. At present, there is a lack of nationwide data regarding hypertension prevalence in Chinese cities. Domestic and international literature searches found only one recent review, which focused on prehypertension in China [40]. Additionally, the latest nationwide investigation of hypertension in China is now ten years old [9]. Therefore, our meta-analysis is relevant to current healthcare needs and is based on a large number of subjects, providing a reliable estimate.

This meta-analysis indicates that >1 in 5 (21.5% (19.4%, 23.6%)) Chinese city residents older than 15 years is hypertensive. Prevalence of hypertension has been on the rise in recent decades, national sampling studies from 1958–1959, 1979–1980, 1991 and 2002 [41]–[43] reported prevalences of 5.1%, 7.7%, 13.7% and 17.6% respectively. Although varying methodologies make comparisons complex, the values from our study exceeded these previous national surveys. Possible explanations for this increase in hypertension prevalence may include economic development, urbanization, aging, lifestyle changes, changing eating habits and deterioration of ecological environments. Furthermore, there has been an increase in overweight and obese individuals. Dyslipidemia, high salt diets, smoking, excessive drinking and sedentary lifestyles all increase risk for hypertension [44]–[52]. It was reported that the percentage of the Chinese population living in urban areas increased from 27% in 1990 to 40% in 2005 [53]. Given this trend, the urban proportion of the population will continue to increase. Effective treatment and prevention measures focusing on the high-risk urban populations will have a profound and favorable impact on public health.

### The Factor of the Geographic Areas

An epidemiological study of 14 provinces reported that hypertension prevalence was higher in northern China than Southern China [54]; our study confirmed this result. The pooled prevalence of hypertension in northern cities is higher than in southern cities, 25.8% and 20.4%, respectively.

### The Factor of Sex

Previous studies have found rates of hypertension to be higher in men than in women at younger ages, while the reverse was true in older participants [55], [56]. Although our study cannot show any information about age groups, it showed that the prevalence of hypertension is higher in men than in women in general, 22.2% and 19.9% respectively. Frank H. Fu and Lena Fung found that female subjects generally have fewer CHD risk factors and thus better health than male subjects [57]. XJ Meng found that urban adults males in China had higher prehypertension, with a prevalence of 47.7% and 33.6% in men and women, respectively [58].

### The Factor of City Sizes

City-size-specific estimates indicate that medium-sized cities have the highest prevalence of hypertension (24.6%), followed by small cities (20.6%), and large cities having the lowest prevalence (18.9%). A cohort study found that hypertension was more prevalence in the more urbanized areas, and inequalities across areas at different stages of urbanization have narrowed [59].

### The Factor of Study Years

The pooled age-standardized prevalence of hypertension is slightly lower among studies performed in 2007–2011 than 2002–2006, 20.6% and 21.9% respectively. Due to the heterogeneity in these studies, a national epidemiology survey should be conducted to confirm if the prevalence of hypertension is truly declining.

### Limitations

While we tried our best to adhere to the guidelines for reporting meta-analysis of observational studies [60], there were several limitations that merit attention.

### Heterogeneity

Meta-regression was not performed, because our study was only designed to report the pooled prevalence in general and in each subgroup. Within each subgroup the heterogeneity of prevalence was high, as such, we assumed that there were other factors likely influencing heterogeneity, including genetic factors, environment, smoking, physical activity and salt intake [61]–[66]. Unfortunately, due to insufficient data in most studies of hypertension prevalence, it is not possible to deduce the effect of these variations on pooled prevalence.

### Measurement Errors

Although strict inclusion and exclusion criteria were used to identify studies in the literature, both inter- and intra-study measurement errors in the ascertainment of BP, classification of subjects and other indices will have occurred. For example, the number of BP measurements can influence the classification of patients as hypertensive [67]. However, in our selected studies, eleven (40.7%) did not reported the time of day that BP measurements were taken or were taken less than three times.

### Representativeness

Because there were great disparities in the distribution of healthcare resources between cities, rural areas, coastal regions and inland regions in China [68], many low-income cities lacked resources to conduct a survey. Although we restrict sampling methods in inclusion criteria, the investigation bias may still affect the meta-analysis outcomes.

In sum, as our study did not identify all variations present in the pooled prevalence. Therefore, we should be careful in prescribing direct policy recommendations from this meta-analysis alone. However, our findings do reveal an important current healthcare issue which deserves the attention of policy makers.
